# Physiological Mechanisms Underlying Tassel Symptom Formation in Maize Infected with *Sporisorium reilianum*

**DOI:** 10.3390/plants13020238

**Published:** 2024-01-15

**Authors:** Yuhe Wang, Chuzhen Xu, Yansong Gao, Yanhua Ma, Xiaoming Zhang, Lin Zhang, Hong Di, Jinxin Ma, Ling Dong, Xing Zeng, Naifu Zhang, Jiawei Xu, Yujuan Li, Chao Gao, Zhenhua Wang, Yu Zhou

**Affiliations:** 1 Key Laboratory of Germplasm Enhancement, Physiology and Ecology of Food Crops in Cold Region, Engineering Technology Research Center of Maize Germplasm Resources Innovation on Cold land of Heilongjiang Province, Northeast Agricultural University, Harbin 150030, China; 2 Institute of Forage and Grass land Sciences, Heilongjiang Academy of Agricultural Sciences, Harbin 150086, China; mayanhua1234@163.com

**Keywords:** maize, head smut, *Sporisorium reilianum*, tassel symptoms

## Abstract

Head smut is a soil-borne fungal disease caused by *Sporisorium reilianum* that infects maize tassels and ears. This disease poses a tremendous threat to global maize production. A previous study found markedly different and stably heritable tassel symptoms in some maize inbred lines with Sipingtou blood after infection with *S. reilianum*. In the present study, 55 maize inbred lines with Sipingtou blood were inoculated with *S. reilianum* and classified into three tassel symptom types (A, B, and C). Three maize inbred lines representing these classes (Huangzao4, Jing7, and Chang7-2, respectively) were used as test materials to investigate the physiological mechanisms of tassel formation in infected plants. Changes in enzyme activity, hormone content, and protein expression were analyzed in all three lines after infection and in control plants. The activities of peroxidase (POD), superoxide dismutase (SOD), and phenylalanine-ammonia-lyase (PAL) were increased in the three typical inbred lines after inoculation. POD and SOD activities showed similar trends between lines, with the increase percentage peaking at the V12 stage (POD: 57.06%, 63.19%, and 70.28% increases in Huangzao4, Jing7, and Chang7-2, respectively; SOD: 27.01%, 29.62%, and 47.07% in Huangzao4, Jing7, and Chang7-2, respectively. These were all higher than in the disease-resistant inbred line Mo17 at the same growth stage); this stage was found to be key in tassel symptom formation. Levels of gibberellic acid (GA_3_), indole-3-acetic acid (IAA), and abscisic acid (ABA) were also altered in the three typical maize inbred lines after inoculation, with changes in GA_3_ and IAA contents tightly correlated with tassel symptoms after *S. reilianum* infection. The differentially expressed proteins A5H8G4, P09233, and Q8VXG7 were associated with changes in enzyme activity, whereas P49353, P13689, and P10979 were associated with changes in hormone contents. Fungal infection caused reactive oxygen species (ROS) and nitric oxide (NO) bursts in the three typical inbred lines. This ROS accumulation caused biofilm disruption and altered host signaling pathways, whereas NO signaling triggered strong secondary metabolic responses in the host and altered the activities of defense-related enzymes. These factors together resulted in the formation of varying tassel symptoms. Thus, interactions between *S. reilianum* and susceptible maize materials were influenced by a variety of signals, enzymes, hormones, and metabolic cycles, encompassing a very complex regulatory network. This study preliminarily identified the physiological mechanisms leading to differences in tassel symptoms, deepening our understanding of *S. reilianum*-maize interactions.

## 1. Introduction

The biotrophic fungus *Sporisorium reilianum* is the causative agent of head smut in maize (*Zea mays* L.). Head smut is a soil-borne disease that can lead to severe yield losses [1]. Since the 1970s, head smut has been a critically important agricultural disease in the United States, Mexico, Australia, South Africa, and France, with incidence rates of up to 80% in some areas [2,3]. Head smut is more devastating to plants when it occurs in areas with low temperatures or high latitudes. In northern China, the incidence of head smut varies from 7% to 35% [4]. Compared to chemical fungicide application or other field management practices, development of smut-resistant varieties is a more convenient, economical, and environmentally sustainable method of preventing this disease. However, a thorough understanding of the mechanisms underlying maize head smut formation is necessary to enable smut resistance breeding.

Fungal pathogens can severely impact plant development. Among maize plants infected with *S. reilianum*, symptoms mainly appear in the inflorescences; the tassels and ears are typically replaced (partially or completely) with large white galls containing dark brown spores. In addition, infection causes a variety of morphological deformities, such as stunted growth, the loss of apical dominance, and the development of additional tillers [5,6]. A detailed investigation of symptom formation could provide new insights into the molecular mechanisms of plant–pathogen interactions, contributing to the development of methods for preventing disease occurrence [7].

Maize plants infected with *S. reilianum* show increased reactive oxygen species (ROS) contents and the altered activities of defense-related enzymes, which in turn activate the maize defense system. He et al. (2006) [8] found that the activities of defense enzymes such as phenylalanine-ammonia-lyase (PAL) (closely related to plant resistance to adversity stress and disease resistance), superoxide dismutase (SOD) (it plays a crucial role in the oxidative and antioxidant balance of the body and is inextricably linked to the onset and progression of many diseases), and polyphenol oxidase (PPO) (PPO protects cells from pathogens by catalyzing the formation of lignin and quinones, which constitute a protective shield, or it can play a direct role in disease resistance through the formation of quinones) are increased to varying degrees in maize after infestation with *S. reilianum*. Changes in enzyme activity are most notable for PAL, which has great physiological significance in enhancing host resistance. In addition, some previous studies have shown that changes in maize hormone contents after infection with *S. reilianum* may be one of the most important factors leading to deformities in head smut-susceptible varieties. For instance, reductions in gibberellic acid (GA_3_) tend to cause plant dwarfing, and decreases in indole-3-acetic acid (IAA) lead to disorganization in flower morphology among susceptible tassels [5,9]. Using an enzyme-linked immunosorbent assay (ELISA) approach (in ELISA, the detection of antigen-antibody reactions relies on enzyme markers. Enzymes can directly label hormone molecules called enzyme-labeled phytohormones. By determining the amount of enzyme-labeled hormone that is bound, the amount of hormone in the unknown sample can be converted), Gao et al. (2011) found that maize plants inoculated with *S. reilianum* exhibit decreases in levels of hormones such as GA_3_ and IAA but significant increases in abscisic acid (ABA). ABA is an important plant stress signaling factor that is closely related to the activities of POD (a class of oxidizing enzymes widely existed in plants with the dual role of eliminating H_2_O_2_ and phenolic amine toxicity) and SOD [10]. Thus, complex interactions between maize and *S. reilianum* together determine maize symptom formation.

In the present study, we assessed tassel symptoms in a total of 55 maize inbred lines containing the Sipingtou bloodline after inoculation with *S. reilianum*. The main symptoms of head smut include the deformation and swelling of the ear with *S. reilianum* and different symptoms of tassel [5,11]. Plants were classified by the phenotype of tassel at the end of the R3 stage based on the presence or absence of original form and function, leafy tufts, and carbonization. Changes in enzyme activities, hormone contents, and protein expression after inoculation were analyzed in three typical inbred lines (Huangzao4, Jing7, and Chang7-2) that showed three different types of tassel symptoms in field trials, respectively. These data were used to explore tassel symptom formation mechanisms at the physiological, biochemical, and molecular levels. In the future, it can be combined with genome-wide association studies, molecular markers, and gene editing technology for disease resistance breeding work [12,13], providing a theoretical basis and germplasm resources for maize resistance to *S. reilianum*.

## 2. Materials and Methods

### 2.1. Maize Materials and S. reilianum Strains

Fifty-five maize inbred lines containing the Sipingtou bloodline and the disease-resistant inbred line Mo17 were provided by several institutes: the Maize Research Institute of the Suihua Branch of the Heilongjiang Provincial Academy of Agricultural Sciences; the Maize Research Institute of the Keshan Branch of the Heilongjiang Provincial Academy of Agricultural Sciences; the Maize Research Institute of the Shandong Provincial Academy of Agricultural Sciences; and the Maize Research Institute of the Northeast Agricultural University. Of the 55 maize inbred lines, Huangzao4, Jing7, and Chang7-2, which belong to the Sipingtou group, were specifically selected for detailed analysis due to their variations in tassel symptoms. Huangzao4, which is sensitive to head smut, is widely used in China and was selected from the natural mixed lines of Sipingtou. Jing7 and Chang7-2 were selected from progeny derived from Huangzao4 as the matrilineal ancestor. Mo17, which is highly resistant to head smut, is a maize inbred line derived from the Lancaster inbred line from the United States.

*S. reilianum* was collected from typical diseased plants at the Xiangyang experimental base of the Northeast Agricultural University in Harbin. Samples were air-dried, then stored at 20 °C in the dark. Teleutospores were removed by passing the samples through a 40-mesh screen [14].

### 2.2. Artificial Inoculation and Phenotypic Analysis

Following the inoculation method of Wang et al. (2015) [15], soil samples were passed through a 2 × 2 mm sieve, then baked in an oven at 105 °C for 4–6 h. Water was added to a final moisture content of 20%. Seeds were sterilized with 1% sodium hypochlorite for 20 min, rinsed five times with distilled water, followed by 4 h in a 37 °C water bath. Sterilized seeds were planted in paper tubes with 5 cm of wet soil at the bottom. Seed embryos were placed face-down on the soil, then covered with ~2 g of 1% *S. reilianum* mycorrhizal soil. Finally, these were covered with ~2 cm of wet soil (20% moisture). Seedlings were cultivated in an artificial climate chamber at 20/15 °C under an 8/16 h light/dark photoperiod. At the V1 stage, the seedlings were moved to the field at the Xiangyang experimental base of the Northeast Agricultural University. Phenotypic surveys were conducted on each plant at the R3 stage (based on the presence or absence of original form and function of the tassel, leafy tufts, and carbonization; R3: milk stage), with photographs taken for documentation.

### 2.3. Enzyme Activity Assays

The maize inbred lines Huangzao4, Jing7, and Chang7-2 and the disease-resistant line Mo17 were inoculated with *S. reilianum* as described above. An uninoculated control was grown under the same conditions. For each line, 0.5 g tassel samples were collected at the VE, V2, V4, V6, V8, V10, V12, and VT stages (five mixed samples. VE: emergence; V1: firstleaf; V(n): nth leaf; VT: tasseling). Crude enzyme solution was extracted as described by Wang et al. (2015) [16], triplicates of each treatment and the activities of POD, SOD, catalase (CAT), PAL, and PPO were quantified as described below.

POD activity. Reaction solution was prepared by dissolving 28 μL of guaiacol (2-methoxyphenol) in 50 mL of 100 mM phosphate-buffered saline (PBS) (pH 6.0) with heating and stirring. After the solution was cooled, 19 μL of 30% H_2_O_2_ was added and mixed well. This reaction mixture was stored at 4 °C prior to use. For each plant sample, 3 mL of reaction mixture solution was combined with 1 mL of crude enzyme solution (or distilled water for the blank control). Optical density (OD) values were measured at 470 nm at 0 min and again at 3 min. The formula used for calculating POD activity was as follows:

$$\mathrm{POD}=\frac{∆A\times V_{t}}{F_{w}\times V_{s}\times t\times 0.1}$$

where V_t_ is the total volume of crude enzyme solution (10 mL), V_s_ is the volume of crude enzyme solution measured (1 mL), ∆A is the change in OD between 0 and 3 min, F_w_ is the fresh sample weight (0.5 g), and t is the reaction time (3 min).

SOD activity. Seven test tubes were set up: three light control tubes, one dark control tube, and three sample tubes (biological replicates). Sample tubes were kept under light conditions at 32 °C for 15 min; the dark control was shaded. After the reaction was completed, the OD values were quickly determined at 560 nm, with the dark control measured first for normalization. SOD activity was calculated as follows:

$$\mathrm{SOD}=\frac{\left(A_{0}-A_{s}\right)\times V_{t}\times 60}{A_{0}\times 0.5\times F_{w}\times V_{s}\times t}$$

where A_0_ is the OD of the light control tube, A_s_ is the OD of the sample tube, V_t_ is the total volume of the extracted crude enzyme solution (10 mL), V_s_ is the volume of enzyme solution measured (0.1 mL), t is the reaction time in the light incubator (15 min), and F_w_ is the fresh sample weight (0.5 g).

PPO activity. Phosphate buffer (1.5 mL at pH 7.2) was combined with 1.5 mL catechol solution and 2.0 mL crude enzyme solution. Samples were heated at 40 °C for 1 h. Reactions were terminated with the addition of 25 µL of 16 mol/L HCl. OD values were measured at 525 nm with a spectrophotometer, with an OD of 0.01 corresponding to 1 unit of enzyme activity. Measurements were taken three times for each sample. PPO activity was calculated as follows:

$$\mathrm{PPO}=\frac{∆A\times V_{t}}{F_{w}\times V_{s}\times t\times 0.01}$$

where V_t_ is the total volume of crude enzyme solution (10 mL), V_s_ is the volume of crude enzyme solution measured (2 mL), ∆A is the change in OD over the measurement time, F_w_ is the fresh sample weight (0.5 g), and t is 1 h.

PAL activity. The method described by [17] was used, with an OD value of 0.01 considered 1 unit of enzyme activity. PAL activity was calculated as follows:

$$\mathrm{PAL}=\frac{∆A\times V_{t}}{F_{w}\times V_{s}\times t\times 0.01}$$

where V_t_ is the total volume of crude enzyme solution (10 mL), V_s_ is the volume of crude enzyme solution measured (2 mL), ∆A is the change in OD over the measurement time, F_w_ is the fresh sample weight (0.5 g), and t is 1 h.

CAT activity. Three sample tubes (biological replicates) and one control tube were tested. Each contained 1.5 mL phosphoric acid buffer, 0.2 mL enzyme extract (boiled crude enzyme for the control), and 1.0 mL distilled water. Each tube was placed in a water bath at 25 °C for 3 min, then 0.3 mL 0.1 mol/L H_2_O_2_ was added. OD_240_ values were measured and CAT activity was calculated as follows:

$$\mathrm{CAT}=\frac{∆A\times V_{t}}{0.1\times V_{s}\times t\times \mathrm{Fw}}$$

where ∆A is the change in OD over the measurement time; F_w_ is the fresh sample weight (0.5 g); V_t_ is the total volume of crude enzyme solution (10 mL); V_s_ is the volume of crude enzyme solution measured (0.2 mL); and t is the reaction time (4 min).

### 2.4. Endogenous Hormone Assay

Samples were collected at four reproductive stages (V4, V8, V12, and VT) as described above for hormone testing. For each sample, 1 g of material was added to 4 mL of cold 80% methanol and 10 ppm antioxidant, ground in liquid nitrogen, then incubated at 4 °C for 24 h to extract endogenous phytohormones. Samples were centrifuged at 8000 rpm and 4 °C for 10 min and the supernatant was collected. Cold 80% methanol (4 mL) was added to the residue and shaken for several hours, then samples were centrifuged using the same parameters. The two supernatants from each sample were mixed and dried under a stream of 99% nitrogen to get the sediment. The sediment was then dissolved in methanol and dilute to volume (2 mL), then filtered through a 0.22 μm nylon membrane for analysis with liquid chromatography (LC) [18,19]. The mobile phase A was methanol and the mobile phase B was aqueous acetic acid (pH 3.6). The injection volume was 10 μL. From 1–30 min, the percentage of mobile phase A was increased from 20 to 60%. The flow rate was 1 mL/min and the detection wavelength was 254 nm. IAA, ABA, and GA_3_ standards were obtained from Shanghai yuanye Bio-Technology Co., Ltd. (Shanghai, China).

### 2.5. Protein Expression Assays

Protein extraction and processing. Maize tassels were collected at the V12 stage using the sampling method described above. Total maize proteins were extracted as described by [20]. Endosperm proteins were extracted with phenol and precipitated with acetone in three repetitions. Total protein was quantified and digested in solution with trypsin as described by [21].

LC–mass spectrometry (MS) detection. The mobile phase A consisted of 0.1% formic acid and 2% aqueous acetonitrile, and the mobile phase B was 0.1% formic acid and 98% aqueous acetonitrile. The flow rate was 300 nL/min. A maize database was queried to identify peaks identified in the raw MS files using MaxQuant (v1.5.2.8). Data vacancy value filling, normalization, and differential expression screening (*p* < 0.05) were conducted in Perseus.

## 3. Results

### 3.1. Differences in Tassel Symptoms between Maize Lines

**Figure 1:**
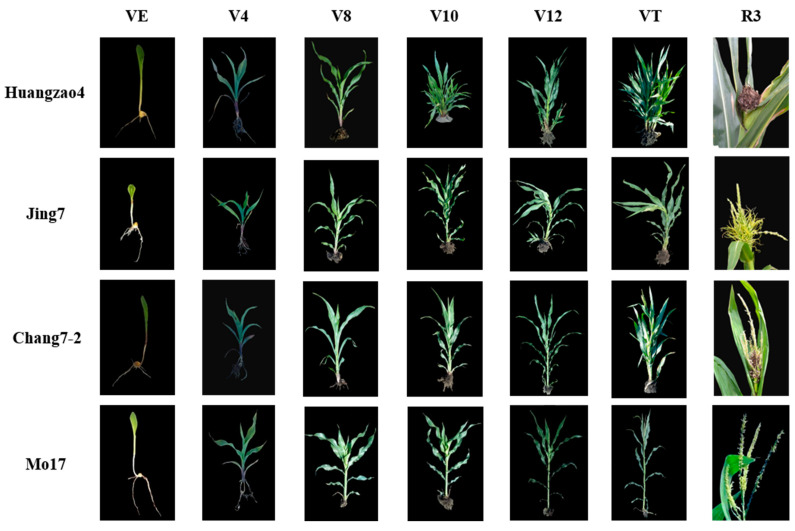
Characters and growth patterns of three typical inbred lines infected with *S. reilianum* Note: the rows indicate the name of the three inbred lines and the columns indicate different growth stages.

After infection with *S. reilianum*, the 55 maize inbred lines showed wide variations in morphology during tassel maturation. The phenotypes were divided into three classes, A–C. Class A showed carbonization of the entire tassel, which had a black, filamentous appearance with a large number of spores. These tassels had completely lost their original form and function. Lines falling into this category included Huangzao4 (Figure 1 column R3), Ji 853, and 434. Class B tassels were swollen and deformed throughout, forming leafy, tufted structures without spores. Lines in this class included Jing7 (Figure 1 column R3), K12, and 444. Class C tassels showed prominent symptoms at the base and a large number of spores, but the upper component continued to develop normally. Plants in this class included Chang7-2 (Figure 1 column R3), Sui line 601, and Csyn 5-4-4. Among the 55 inbred lines containing Sipingtou blood, the symptoms of lines Huangzao4, Jing7, and Chang7-2 were the most typical of classes A, B, and C, respectively.

Plant morphology was evaluated at multiple growth stages after inoculation with *S. reilianum* in the three typical inbred lines (Huangzao4, Jing7, and Chang7-2) and in the disease-resistant control inbred line Mo17. The latter had stout stalks and showed no disease symptoms in the VE–VT stages. Huangzao4, Jing7, and Chang7-2 leaves showed regressed green spots at the VE–V8 stages, but there were no significant differences in symptoms between lines. At the V10 stage, Huangzao4 showed significantly repressed growth; the internodes were shortened and the plants were dwarfed. This was in contrast to Jing7 and Chang7-2, which showed no significant differences in growth. The terminal leaves of Jing7 appeared to twist and droop in the V12 stage, although there were no obvious changes in this type in Huangzao4 or Chang7-2. In the VT stage, Huangzao4 plants did not generate normal tassels; the tassel structure was totally destroyed, carbonized, and filamentous. Jing7 tassels began to form a leafy structure from the bottom, which was swollen and had no obvious spores on the surface. In Chang7-2, a small number of spores or spore clusters appeared at the bottoms of the tassels, but the overall tassel structure was normal (Figure 1 column VE–VT). These results indicated that the V12–VT stages were crucial for tassel symptom formation. External morphology demonstrated that Huangzao4 was infected by *S. reilianum* at an earlier stage and was more severely affected than the other lines; furthermore, symptomatic differences between the three inbred lines were primarily reflected in the tassels.

### 3.2. Enzyme Activity in Infected Tassels

After the artificial inoculation of three typical maize inbred lines and the disease-resistant control inbred line Mo17 with *S. reilianum*, there were variations in the activities of defense-related enzymes in the tassel between different vegetative stages, which may be connected with the formation of different tassel symptoms in maize infected with *S. reilianum*.

**Figure 2:**
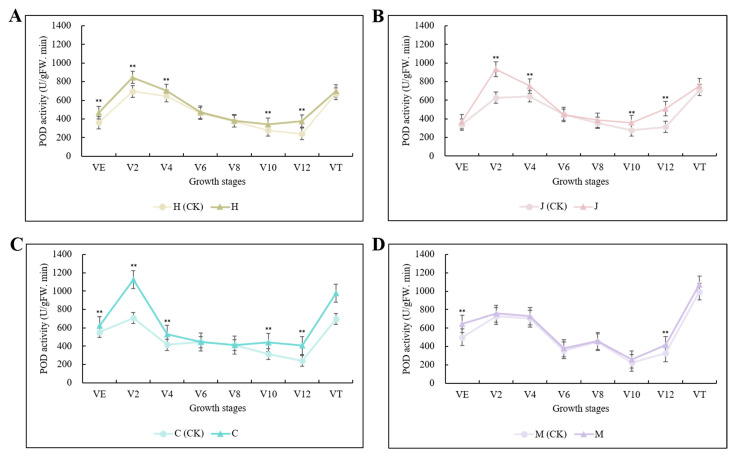
Changes in POD activity in three typical maize inbred lines and disease-resistant control inbred line Mo17 at different growth stages. Note: (**A**): H (CK) indicates Huangzao4-uninoculated plants; H indicates Huangzao4-inoculated plants; (**B**): J (CK) indicates Jing7-uninoculated plants; J indicates Jing7-inoculated plants; (**C**): C (CK) indicates Chang7-2-uninoculated plants; C indicates Chang7-2-inoculated plants; (**D**): M (CK) indicates Mo17-uninoculated plants; M indicates Mo17-inoculated plants; “**” indicates *p* < 0.01.

POD activity was increased in all three typical maize inbred lines after inoculation. Chang7-2 was the most sensitive to infection (i.e., had the highest POD activity), followed by Jing7, then Huangzao4. In all three lines, the increase in POD activity peaked at the V12 stage, with 57.06%, 63.19%, and 70.28% increases in Huangzao4, Jing7, and Chang7-2, respectively (Figure 2A–C); these were all much higher than in the disease-resistant inbred line Mo17 at the same growth stage (27.52%) (Figure 2D). Thus, infection with *S. reilianum* elevated host POD activity, but the extent of this elevation differed between lines with different symptomatic types. Consistent with the observations of tassel morphology, these results suggested that the V12 stage was critical for tassel symptom formation. Furthermore, the sharp rise in POD activity in Chang7-2 may have been associated with its maintenance of structural integrity in the tassel.

SOD activity was increased after inoculation in all three typical maize inbred lines, showing similar trends to those of POD activity. Increases in SOD activity also peaked at the V12 stage, with increases of 27.01%, 29.62%, and 47.07% in Huangzao4, Jing7, and Chang7-2, respectively (Figure S2A–C). Again, these were all higher than in the disease-resistant inbred line Mo17 at the same growth stage (16.65%). In Mo17, SOD activity peaked at the VE stage, then gradually decreased (Figure S2D). Overall, infection with *S. reilianum* induced SOD activity in the host, and the V12 stage appeared to be associated with tassel symptom formation. The similar trends in SOD and POD activities indicated that these enzymes may have been jointly involved in the resistance of disease-susceptible plants to ROS damage. Furthermore, the antioxidant defense capacity of Chang7-2 was determined to be higher than that of Jing7 or Huangzao4.

After Huangzao4 inoculation with *S. reilianum*, PPO activity increased steadily in the early stages, then decreased in the VT stage. In Jing7, PPO activity increased dramatically in the early stages, then decreased significantly from V12–VT (*p* < 0.01). In contrast to both of those lines, PPO activity was consistently elevated in Chang7-2 (Figure S3A–C). These results showed that infection with *S. reilianum*-altered PPO activity in all susceptible inbred lines, but that the three typical maize inbred lines differed in their responses. Specifically, the decline in PPO activity among Huangzao4 and Jing7 plants was likely related to the complete destruction of the tassel cellular structure.

After inoculation, increases in PAL activity were not statistically significant in any of the susceptible lines at the VE stage. However, PAL activity continued to increase, peaking at different stages between the three lines. The maximum increase in PAL activity was observed in Huangzao4 at the V10 stage, with an increase of 28.39% (Figure S4A). In contrast, PAL activity peaked in Jing7 and Chang7-2 at the V12 stage, with increases of 67.18% and 51.55%, respectively (Figure S4B,C). Thus, infection with *S. reilianum* consistently increased host PAL activity, but there were differences between the three typical maize inbred lines. PAL can control the synthesis of a variety of antimicrobial substances and thus likely affected tassel symptom formation extensively.

All three susceptible inbred lines showed decreases in CAT activity after inoculation, whereas there were no significant changes in Mo17 at any growth stage (Figure S5). Thus, *S. reilianum* did not cause a strong CAT reaction. Low CAT activity at later stages of infection may have been associated with peroxisome destruction caused by the pathogen.

Overall, we here found that antioxidant enzyme activities at several key growth stages (V10, V12, and VT) were essential for the formation of tassel symptoms. At the V10 stage, which primarily involved the PAL response, Huangzao4 had the strongest response. The VT stage mainly involved PPO activity, which decreased in both Huangzao4 and Jing7 during this period but increased in Chang7-2. The V12 stage primarily involved the POD and SOD responses, with the increase in both enzymes peaking in this period. Line-specific differences in antioxidant enzyme activities at these stages were closely associated with phenotypic differences in tassel symptoms.

### 3.3. Changes in Endogenous Hormones Related to Tassel Symptoms

**Figure 3:**
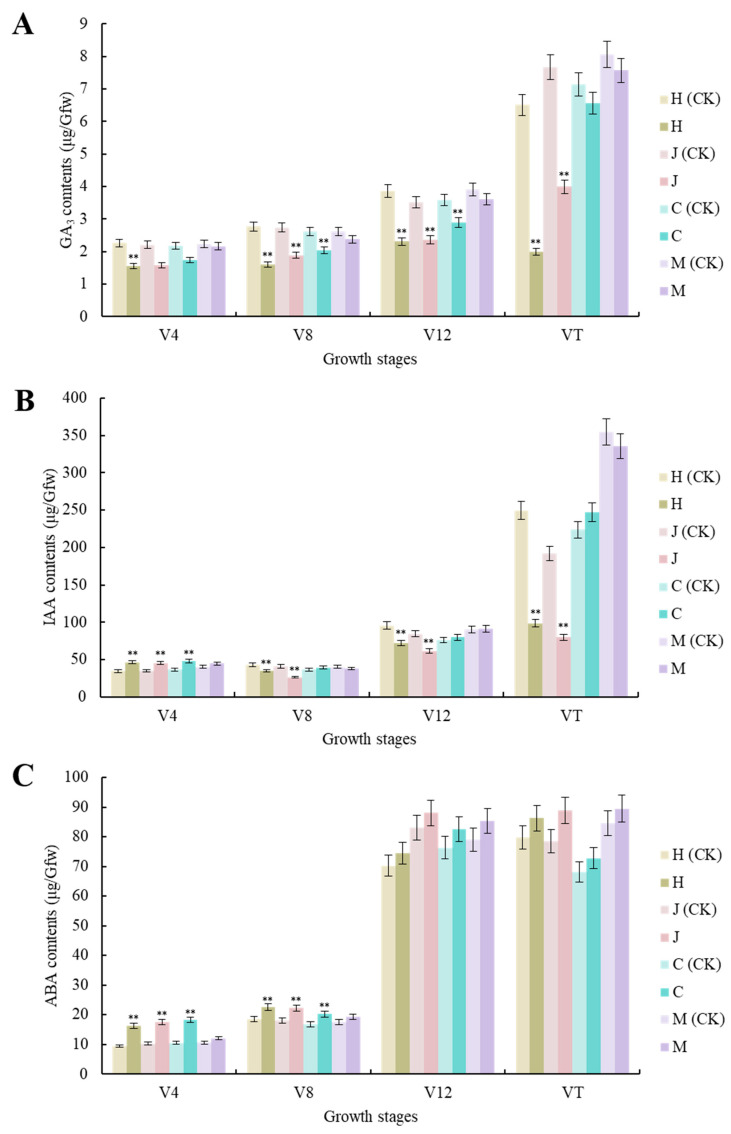
Changes in contents of three different hormones in three typical maize inbred lines and disease-resistant control Mo17 at different growth stages. (**A**) Changes in GA_3_ contents in three typical maize inbred lines and resistant inbred line Mo17 at different growth stages. (**B**) Changes in IAA contents in three typical maize inbred lines and resistant inbred line Mo17 at different growth stages. (**C**) Changes in ABA contents in three typical maize inbred lines and resistant inbred line Mo17 at different growth stages. Note: H (CK) indicates Huangzao4-uninoculated plants; H indicates Huangzao4-inoculated plants; J (CK) indicates Jing7-uninoculated plants; J indicates Jing7-inoculated plants; C (CK) indicates Chang7-2-uninoculated plants; C indicates Chang7-2-inoculated plants; M (CK) indicates Mo17-uninoculated plants; M indicates Mo17-inoculated plants; “**” indicates *p* < 0.01.

Compared with the uninoculated control, the GA_3_ contents of the three typical maize inbred lines were significantly decreased (*p* < 0.01) at the V4, V8, and V12 stages. There were also significant decreases (*p* < 0.01) in Huangzao4 and Jing7, but not Chang7-2, at the VT stage. There were no significant changes in GA_3_ contents in the disease-resistant inbred line Mo17 at any growth stage. Huangzao4 showed the strongest decrease in GA_3_ contents, followed by Jing7, then Chang7-2 (Figure 3A). Phenotyping revealed that Huangzao4 was severely dwarfed, Jing7 was mildly dwarfed, and the Chang7-2 height was unchanged. This indicated a potentially close relationship between decreases in GA_3_ contents and internode shortening.

There were no significant changes in the IAA contents of the disease-resistant inbred line Mo17 at any growth stage after inoculation. The three typical maize inbred lines showed similar changes (significant increases) at the V4 stage (*p <* 0.01). The V8–VT period showed similar trends and continued declines among Huangzao4 and Jing7 plants. This may have accounted for the disordered tassel morphology of these plants in the later period. In contrast, Chang7-2 showed statistically insignificant changes, which could explain the normal flower morphology in this line (Figure 3B).

Compared with the uninoculated control, there were significant increases in ABA content in all three typical maize inbred lines at the V4 and V8 stages (*p* < 0.01 and *p* < 0.05, respectively) but no significant increases at the V12–VT stages. The disease-resistant inbred line Mo17 showed non-significant changes in ABA contents across all growth periods (Figure 3C). These results indicated that infection with *S. reilianum* tended to increase ABA contents and that the three maize inbred lines with different tassel symptoms showed very similar trends in ABA levels across growth periods; this suggested that the differences in overt symptoms were not primarily due to differences in ABA contents.

Overall, the analysis of hormone levels showed that, compared with the uninoculated control, there were no significant changes in hormone contents in the disease-resistant control inbred line Mo17 after inoculation. The three typical maize inbred lines did show significantly altered hormone contents after inoculation, with changes in GA_3_ and IAA contents closely associated with differences in tassel morphology after infection with *S. reilianum*. Furthermore, the V12 stage appeared to be a key turning point in determining tassel morphology.

### 3.4. Differentially Expressed Proteins Associated with Differences in Tassel Symptoms

**Figure 4:**
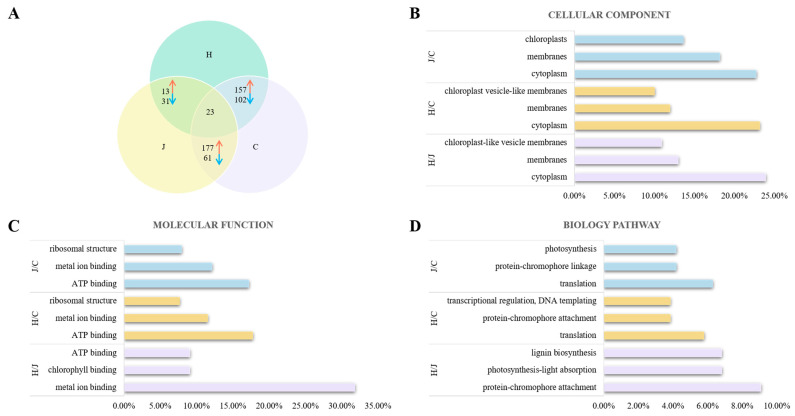
Analysis of differentially expressed proteins in typical maize inbred lines infected with *Sporisorium reilianum* tassels with different symptoms. (**A**) Analysis of differently expressed protein between three types lines with different symptoms in tassel. (**B**) Cellular component analysis of different expressed protein in three types lines with different symptoms in tassel. (**C**) Molecular function analysis of different expressed protein in three types lines with different symptoms in tassel. (**D**) Biology pathway analysis of different protein in three types lines with different symptoms in tassel. Note: Red represents upregulated expressed proteins, blue represents downregulated expressed proteins.

Proteomic analyses were conducted in the tassels of the three typical maize inbred lines to identify proteins that were differentially expressed. At a threshold of *p* < 0.05, there were 44, 259, and 238 differentially expressed proteins between Huangzao4 and Jing7, Huangzao4 and Chang7-2, and Jing7 and Chang7-2, respectively; 23 proteins were differentially expressed in all three comparisons (co-differentially expressed proteins) (Figure 4A). Co-differentially expressed proteins included catalase isozyme 3, superoxide dismutase [Cu-Zn] 4A, isoflavone reductase homolog IRL, phenylalanine/tyrosine ammonia lyase, and other proteins associated with fungal infections.

The differentially expressed proteins were then classified with Gene Ontology (GO) terms to assess their functions. Three types of GO terms were analyzed: cellular components, molecular functions, and biological processes. Cellular component analysis revealed that differentially expressed proteins between Huangzao4 and Jing7 were primarily distributed in the cytoplasm (10 proteins), membranes (eight), and the chloroplasts (six), which accounted for 22.73%, 18.18%, and 13.64%, respectively, of all differentially expressed proteins in this comparison. For Huangzao4 and Chang7-2, differentially expressed proteins were mainly distributed in the cytoplasm (60; 23.17%), membranes (31; 11.97%), and chloroplast vesicle-like membranes (26; 10.04%). In the comparison of Jing7 with Chang7-2, most of the differentially expressed proteins were distributed in the cytoplasm (57; 23.95%), membranes (31; 13.03%), and chloroplast-like vesicle membranes (26; 10.92%). The most common differentially expressed proteins in the cytoplasm among all comparisons were superoxide dismutase, the phenylalanine/tyrosine ammonia-cleaving enzyme, and microtubule proteins (Figure 4B).

With respect to molecular functions, most of the differentially expressed proteins between Huangzao4 and Jing7 showed binding functions, such as metal ion binding (14; 31.82%), chlorophyll binding (4; 9.09%), and ATP binding (4; 9.09%). The most prominent molecular functions of differentially expressed proteins between Huangzao4 and Chang7-2 were ATP binding (46; 17.76%), metal ion binding (30; 11.58%), and ribosomal structure (20; 7.72%). In the comparison of Jing7 and Chang7-2, the most common molecular functions of differentially expressed proteins were ATP binding (41; 17.23%), metal ion binding (29; 12.18%), and ribosomal structure (19; 7.98%) (Figure 4C).

The most highly represented biological process terms among differentially expressed proteins between Huangzao4 and Jing7 were protein–chromophore attachment (4; 9.09%), photosynthesis-light absorption (3; 6.82%), and lignin biosynthesis (3; 6.82%). The most enriched biological process terms among differentially expressed proteins between Huangzao4 and Chang7-2 were translation (15; 5.79%), protein–chromophore attachment (10; 3.86%), transcriptional regulation, DNA templating (10; 3.86%), and other biological processes. For the comparison of Jing7 with Chang7-2, the most abundant biological process terms among differentially expressed proteins were translation (15; 6.30%), protein–chromophore linkage (10; 4.20%), and photosynthesis (10; 4.20%). These results supported the existence of differences in the chloroplasts between the three lines; differences in photosynthetic systems may therefore have been one of the most important factors involved in the formation of different disease-sensitive phenotypes (Figure 4D).

**Figure 5:**
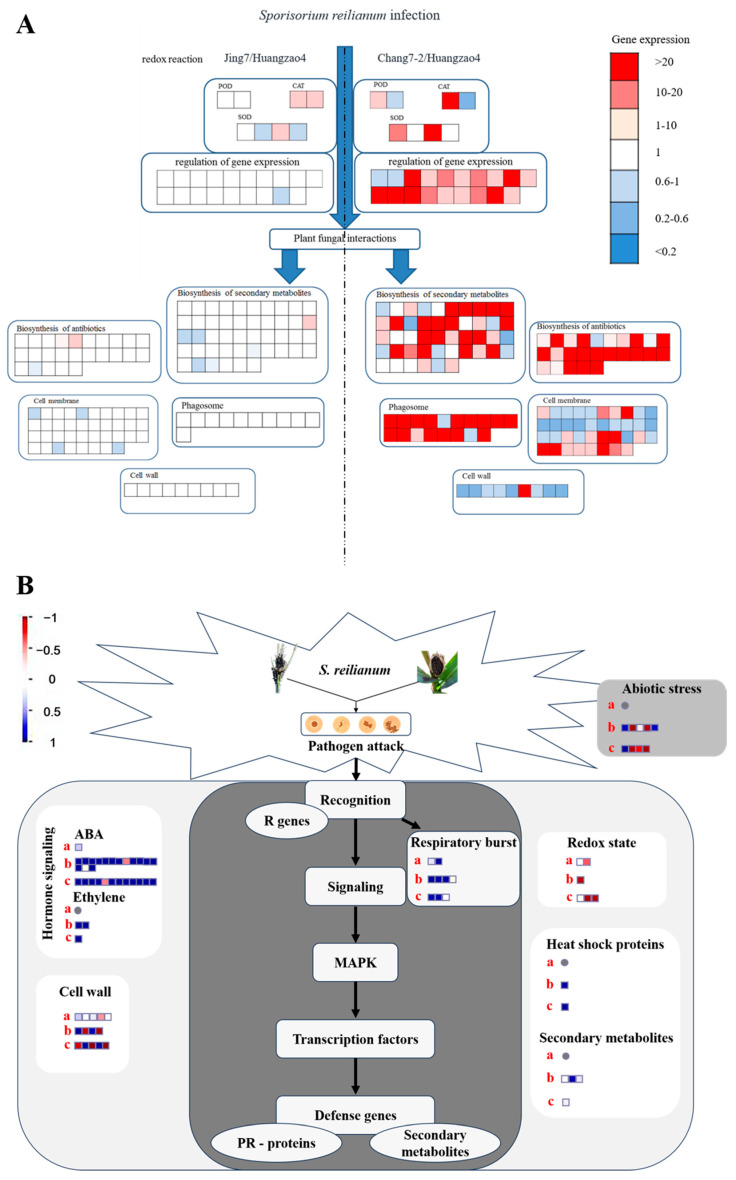
Analysis of proteins and genes involved in the regulation of different tassel symptoms of maize infected with *Sporisorium reilianum.* (**A**) Analysis of different expressed proteins related to different symptoms of tassel. (**B**) Pathways regulating responses to pathogenic fungal *Sporisorium reilianum* stress in maize. Note: (**A**) The amount of gene expression is based on Huangzao4. (**B**) a: Huangzao4 vs. Jing7; b: Huangzao4 vs. Chang7-2; c: Jing7 vs. Chang7-2.

The Kyoto Encyclopedia of Genes and Genomes (KEGG) gene product classification system was used to analyze the metabolic pathways in which differentially expressed proteins were involved. The most highly enriched pathways were metabolism, secondary metabolite synthesis, antibiotic synthesis, and peroxisomes (Figure 5A). Many co-differentially expressed proteins were involved in the peroxisomal pathway, including SOD and CAT regulatory genes such as *SODM1* (P09233), *SODC2* (P11428), *SODC4* (P23345), *SODC5* (P23346), *CATA1* (P18122), and *CATA3* (P18123) [22,23]. Many differentially expressed proteins were also associated with POD regulation, including *PER1* (A5H8G4) and *PER42* (A5H453) [24]. This indicated that antioxidant reductase proteins had important roles in tassel symptom formation processes.

Numerous proteins involved in secondary metabolite synthesis were also differentially expressed between the three typical maize inbred lines. One such protein, PALY (Q8VXG7), is the first key enzyme in phenylalanine metabolism and thus directly affects PAL and the downstream synthesis of phenylalanine analogues, quinones, and flavonoids [25]. In addition, a large number of hormone receptor proteins and proteins associated with hormone synthesis and degradation pathways were differentially expressed. For instance, IAA synthesizes indole-3-acetaldehyde oxidase (which is regulated by *ALDO2*) and auxin-binding protein 1 (regulated by *ABP1*).

The structures of the membranes, cell wall, and autophagosomes, combined with analysis of differentially expressed proteins between Huangzao4 and Jing7, suggested that phenotypic differences between these lines may have been mainly due to teliospore formation. Protein expression in Chang7-2 differed greatly from protein expression in the other two lines, which could be related to the complete male spike damage observed in Huangzao4 and Jing7 but normal tassel development in Chang7-2 after disease onset (Figure 5B). There were also significant differences in plant metabolism, the tricarboxylic acid cycle, glycolysis, carbon metabolism, lipid metabolism, and other processes between Chang7-2 and the other two lines.

## 4. Discussion

Previous research has demonstrated that maize infection with *S. reilianum* leads to flower organ damage, ear shortening and swelling, and the presence of smut spores in the bracts [26]. In severe cases, infected tassels form galls and carry a large number of spores or form leafy clusters without black powder spores [14,27]. Lines such as Mo17, Liao 1311, and B70 show high resistance that is stably heritable; these lines have demonstrated average incidence rates < 2.5% across four to six years of field trials with little variation between years [28]. Mo17 had an average *S. reilianum* disease incidence of just 1.5% in 22 replicate experiments over five years, whereas the sensitive inbred line Huangzao4 had a five-year average incidence rate of 88.7%. Meta-analyses indicate that disease incidence among inbred lines is relatively stable but is slightly affected by parameters such as the year, location, and environmental factors.

Here, we conducted a preliminary multi-year, multi-point analysis of Sipingtou bloodline maize inbred lines after inoculation with *S. reilianum*. We noted stability not only in incidence rates but also in tassel symptoms, which could be divided into three classes: A, B, and C. The inbred lines Huangzao4, Jing7, and Chang7-2 typified classes A-C, respectively. Class A symptoms included a completely charred tassel that produced a large number of teliospores; Class B had an enlarged tassel and no spores; and Class C had a damaged tassel base but a normally developing upper region. This suggested that the symptomatic manifestations of head smut were stably expressed and associated with host genetic factors.

Pathogenic microorganisms can cause specific symptoms in plants, often due to complex plant–pathogen interactions that alter the cellular morphology, growth, and metabolic processes of the host. Vigorous antioxidant enzyme activity is required to neutralize ROS overproduction in host plants and thus prevent extensive cellular damage. Previous studies have found that pathogen infection increases SOD and POD activity in some plants such as *Coffea arabica*, tobacco, and mung bean [29,30,31,32]. Changes in POD and SOD activities in plants are directly related to increases in ROS and cell membrane degradation, which are important indicators of pathogen resistance during the tasseling stage [33,34]. PAL is a key enzyme in the phenylaprapanoid metabolism pathway that participates in the synthesis of key specialized metabolites [35,36]. The elevated activity of PPO, a copper metalloprotein, accelerates oxidation of phenolic compounds, converting them to quinones. Quinones are highly toxic to pathogenic fungi and contribute to host defenses [37,38]. Another antioxidant enzyme, CAT, breaks down excess H_2_O_2_ to protect cells from damage. Previous research from our lab found that maize tassel symptoms primarily occur during the V12–VT stages, during which the cellular structure is completely destroyed and visible teliospores form in the diseased tissues of Huangzao4 and Chang7-2 plants. In Jing7, the cellular contents disappear completely, with only the cell wall remaining and an absence of visible teliospores [39]. Teliospore formation may be induced by host secretions and can damage host cells; thus, differences in teliospore abundance may underlie line-specific differences in tassel symptoms.

Inoculation with *S. reilianum* activates the maize enzymatic defense system. We here found that the activities of POD, SOD, and PAL were increased in the three typical inbred lines after inoculation, with the highest levels in Chang7-2. In contrast, CAT activity decreased after inoculation, which may have been due to peroxisome destruction. In Huangzao4 and Jing7, PPO activity increased in the early stages of growth, but decreased later. In contrast, PPO activity remained consistently high in Chang7-2, which could explain the lack of complete tassel structure destruction in that line.

Endogenous phytohormone levels also play important roles in plant pathogen resistance. Barna et al. (2012) [40] found that hormones strongly influence the production of ROS and antioxidants, which are closely associated with plant pathogen resistance. Prior studies have extensively analyzed the roles of phytohormones such as ethylene, jasmonic acid, and salicylic acid in plant immune system responses to biotic stressors [41,42,43,44,45]. IAA, GA_3_, and cytokinin (CTK) have also been described as regulators of plant pathogen resistance [46,47,48]. Changes in endogenous hormone levels are known to be the main causes of plant dwarfing and the disruption of tassel development after *S. reilianum* inoculation [5,49]. Furthermore, GA is thought to play an important role in sorghum symptom formation in response to *S. reilianum* infection. The presence of this fungus has been shown to decrease host GA concentrations, which may cause increased tillering in infected plants [50]. IAA is an essential hormonal signal that influences floret morphogenesis and regulates plant–pathogen interactions [51]. IAA affects sporangia formation, flower formation [52,53,54], and differences in morphology between male and female tassels [5,19]. We here found reduced GA_3_ contents among the susceptible maize lines after inoculation with *S. reilianum*, with the strongest decrease in Huangzao4. We therefore hypothesized that decreased GA_3_ levels were the primary cause of the significant internode shortening observed in Huangzao4 and of differences in symptom formation between Jing7 and Chang7-2 plants. IAA contents did not change significantly in Chang7-2 after infection, whereas IAA levels decreased markedly in Huangzao4 and Jing7. This could explain the normal upper tassel development in Chang7-2 after head smut onset. We also found that ABA contents increased in maize after *S. reilianum* infection, consistent with the findings of [55]. However, the three typical maize inbred lines were very similar in terms of ABA levels and trends at all growth stages. This result suggests that the increase in ABA content is not the main factor contributing to the differences in symptoms among the lines. Protein expression reflects the true state of a plant cell and is the key link between gene expression and metabolism [56,57]. Here, proteomic analysis showed that differentially expressed proteins between the three susceptible lines were mainly distributed in the cytoplasm and the cell membrane. Both cytoplasm and cell membrane play important roles in maintaining cellular structure and transmitting signals. However, there were differentially expressed proteins in the peroxisomes and specialized metabolite synthesis pathways, indicating that antioxidant enzymes and specialized metabolite synthesis affected disease symptom manifestation. Changes in the cellular redox status are some of the earliest detectable signals in pathogen-challenged cells; a wide variety of enzymes are involved in this pathway [58]. ROS are rapidly produced by host plants when pathogens attempt to invade [59]. High levels of ROS can lead either directly or indirectly to host lipid peroxidation [60], which disrupts normal cellular function and is also thought to be a signal induced by active cells under stressful conditions. Cells are typically protected from ROS by antioxidant systems, both enzymatic and nonenzymatic. ‘Yayu889’ was found to be enriched in four important redox enzymes after infection with gray leaf spot: cytochrome P450, acyl-desaturase 2, geraniol 8-hydroxylase, and indolin-2-one monooxygenase. POD activity is enhanced after the establishment of infection, validating GO term results at the metabolic level [61]. The inhibition of auxin signaling repression weakens *Arabidopsis thaliana* resistance to the necrotrophic fungi *Plectosphaerella cucumerina* and *Botrytis cinerea* [62].

We here found relatively limited differential protein expression between Huangzao4 and Jing7, which may explain the extensive tassel disease state in both varieties. However, the formation of winter spores may be the key to phenotypic differences observed between Huangzao4 and Jing7. A large number of differentially expressed proteins associated with POD and SOD expression were identified between the two lines. Enhanced defense enzyme activities are known to promote host resistance to pathogen-induced damage. Chang7-2 showed more extensive increases in POD and SOD activity than Huangzao4 or Jing7. Furthermore, a sharp increase in POD activity in Chang7-2 plants at the V12 stage may have been associated with its capacity to maintain the tassel structure after disease onset when the other two lines showed completely destroyed tassel structures. There were also differences in the expression of proteins regulating IAA synthesis; Chang7-2 did not show significantly altered IAA contents, whereas the other two showed significantly lower IAA levels at the late growth stages. This could have contributed to the maintenance of normal tassel morphology in Chang7-2.

The results of this study indicated that *S. reilianum* infection in maize increased ROS and NO levels in the three typical maize inbred lines. The NO signals strongly triggered a host-specialized metabolism, whereas ROS accumulation resulted in the destruction of biofilms and nucleic acids; the loss of protein function; the disruption of hormone signals; and host cell apoptosis. The synthesis of antioxidant defense enzymes and antimicrobial substances in Chang7-2-limited pathogen-induced cellular damage, preventing morphological changes to the upper tassel; in contrast, the metabolism was completely disrupted in Huangzao4 and Jing7. These findings indicate that interactions between *S. reilianum* and disease-susceptible materials were affected by a variety of signals, enzyme activities, hormones, and metabolic cycles, representing a highly complex host-regulatory network.

**Figure 6:**
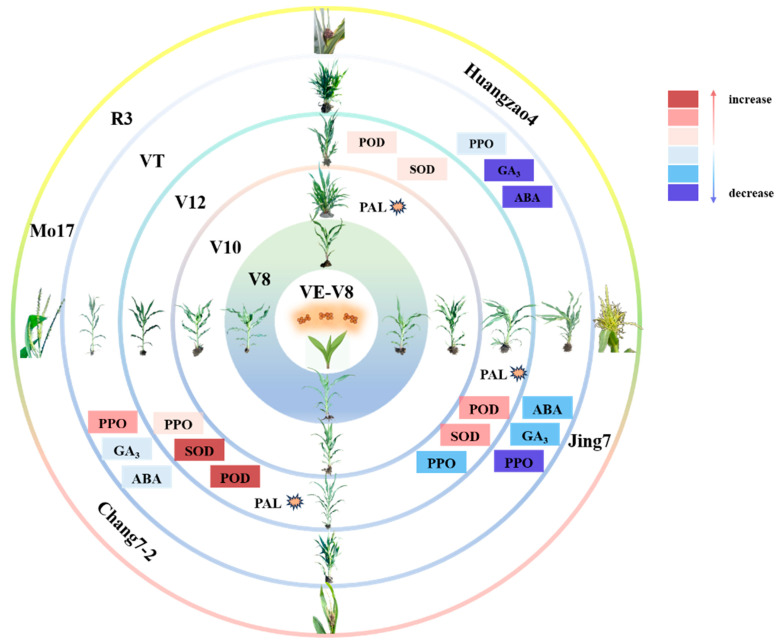
Important enzyme activities and hormonal changes at critical stages of different tassel symptoms formation. Note: The pink series of colors represents the increase, and the lightest to darkest color represents a larger increase; the blue series of colors represents a decrease, and the color from light to dark represents a greater decline; and the explosion symbol represents the enzyme activity increase peaked at this stage.

The V12–VT stage is essential in the formation of tassel symptoms after disease onset in susceptible inbred lines. In a previous study, we found key changes at the V12–VT stage in the inbred lines Huangzao4 and Chang7-2 (cell wall disintegration and intracellular spore production) and Jing7 (complete loss of cell contents with only the cell wall remaining). Spore formation may be induced by host secretions but can cause damage to host cell walls, enabling further pathogen invasion into the plant [39]. Plant–pathogen interactions lead to a variety of changes in host defense-related enzymes and hormone contents. Indeed, we here found differences in the expression of proteins related to defense enzyme activity and hormone contents in the three inbred lines. POD, SOD, and PAL activities peaked in Chang7-2 at this stage, at higher levels than in Huangzao4 or Jing7. The PAL activity of Huangzao4 peaked in increase during V10 before Jing7 and Chang7-2, which may be the cause of Huangzao4’s inability to form a tassel structure. PPO activity also consistently increased in Chang7-2, in contrast to the consistent decreases in the other two lines. These increases in defense-related enzyme activities allowed Chang7-2 plants to minimize the damage from pathogen invasion, alleviate the negative impacts of cell wall structural collapse, and maintain normal tassel structure. Although levels of GA_3_ and IAA decreased significantly in Huangzao4 and Jing7, they were not significantly altered in Chang7-2. Thus, Chang7-2 did not show symptoms of dwarfing (like Huangzao4) or increased tillering (like Jing7). These results demonstrate that a combination of pathogen invasion, hormone levels, enzyme activities, and protein expression during the V12–VT stage led to the formation of different tassel symptoms between the three maize inbred lines (Figure 6).

## 5. Conclusions

Tassel symptoms in inbred Sipingtou maize infected with *S. reilianum* were significantly different between lines and were stably heritable. The three typical inbred lines (Huangzao4, Jing7, and Chang7-2) showed similar trends in POD and SOD activity, with the largest and smallest increases in enzyme activity occurring in Chang7-2 and Huangzao4, respectively. Increases in the activities of these enzymes peaked at the V12 stage, which may have been a critical period for the formation of tassel phenotypes. Changes in GA_3_ and IAA contents were also closely related to tassel symptoms among infected plants, with the V12 stage again serving as a key turning point. The three lines showed a differential expression of proteins related to POD, SOD, and PAL activities (A5H8G4, P09233, and Q8VXG7, respectively) and of proteins related to synthesis or metabolism of GA_3_, IAA, and ABA (P49353, P13689, and P10979, respectively). Overall, our results indicate that the disruption of membrane structure and the synthesis of specialized metabolites due to ROS bursts and NO signaling, respectively, may have driven the development of tassel symptoms in infected maize. These findings enhance our understanding of symptom development in a key plant–pathogen system, ultimately contributing to the identification of strategies to promote plant disease resistance.

This study reveals the critical period of changes in maize defense-related enzyme activities and hormone contents during the infestation of *S. reilianum*, which is closely related to the formation of different symptoms in maize tassels. This will be useful for future metabolomics and transcriptomics studies based on the critical stage, and combined with proteomics results, we will jointly analyze and search for relevant genes regulating maize resistance to *S. reilianum*, as well as clarify the regulatory functions of these candidate genes and obtain disease-resistant varieties through gene editing and other technologies to provide germplasm resources and a theoretical basis for work on maize resistance to head smut breeding.

## Data Availability

Data are contained within the article and Supplementary Materials.

## Supplementary Materials

The following supporting information can be downloaded at: https://www.mdpi.com/article/10.3390/plants13020238/s1.
