# Supplementary material for: Physiological Mechanisms Underlying Tassel Symptom Formation in Maize Infected with Sporisorium reilianum
Source: Plants (Basel). 2024 Jan 15;13(2):238. doi: 10.3390/plants13020238 (PMC10820020; doi:10.3390/plants13020238)
Supplement: Supplementary file 1 [file plants-13-00238-s001.zip › plants-2732331-supplementary-figures.pdf]

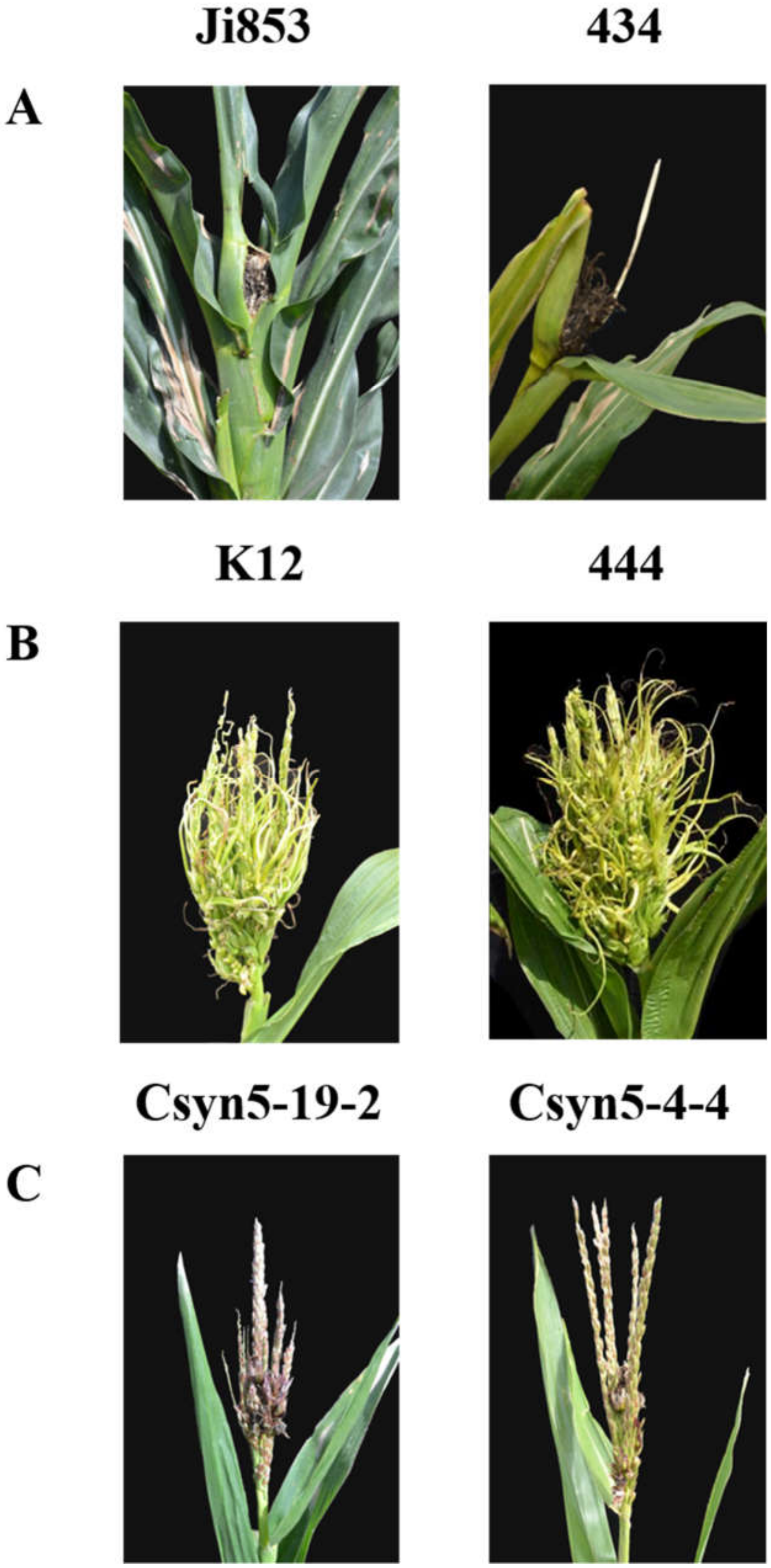

Figure S1. A、B and C type symptoms of tassel of typical maize inbred lines infected with *S. reilianum*
Note: the rows represent A、B and C type and the columns represent the name of the inbred line.

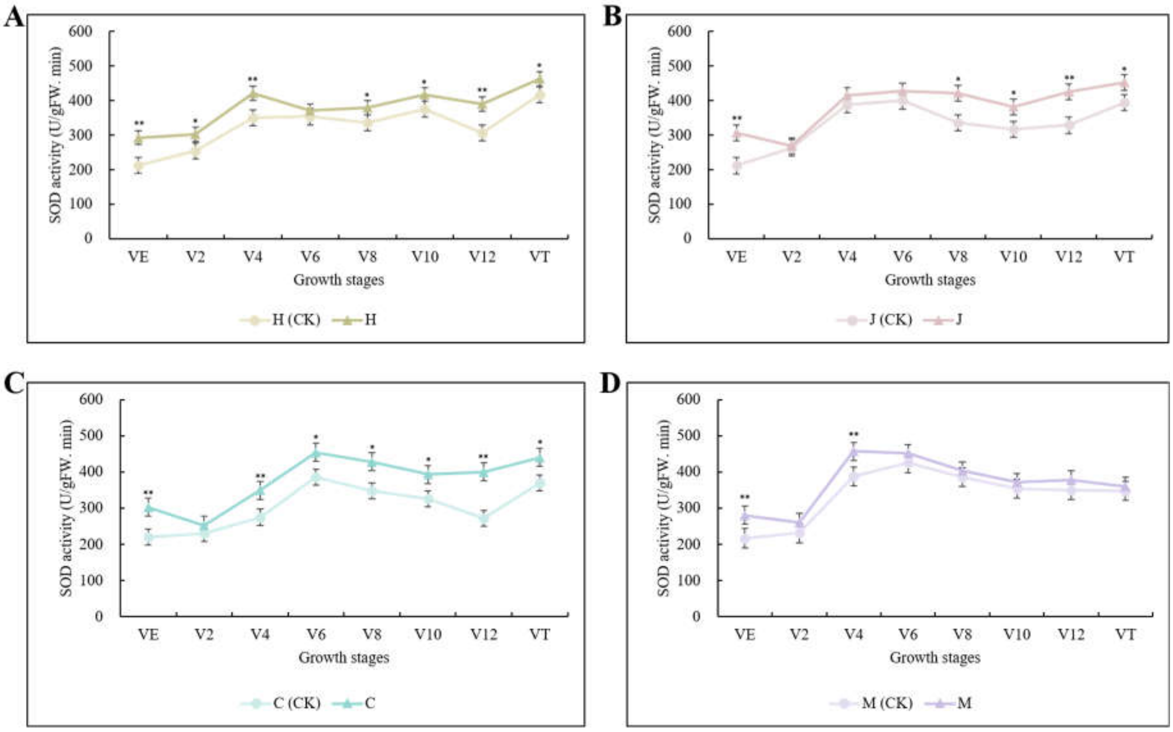

Figure S2. Changes of SOD activitiy in three typical maize inbred lines and disease-resistant control inbred line Mo17 at different growth stages Note: A: H (CK) indicate Huangzao4 uninoculated plants; H indicate Huangzao4 inoculated plants; B: J (CK) indicate Jing7 uninoculated plants; J indicate Jing7 inoculated plants; C: C (CK) indicate Chang7-2 uninoculated plants; C indicate Chang7-2 inoculated plants; D: M (CK) indicate Mo17 uninoculated plants; M indicate Mo17 inoculated plants; "*" indicates $p<0.05$; "**" indicates $p<0.01$.

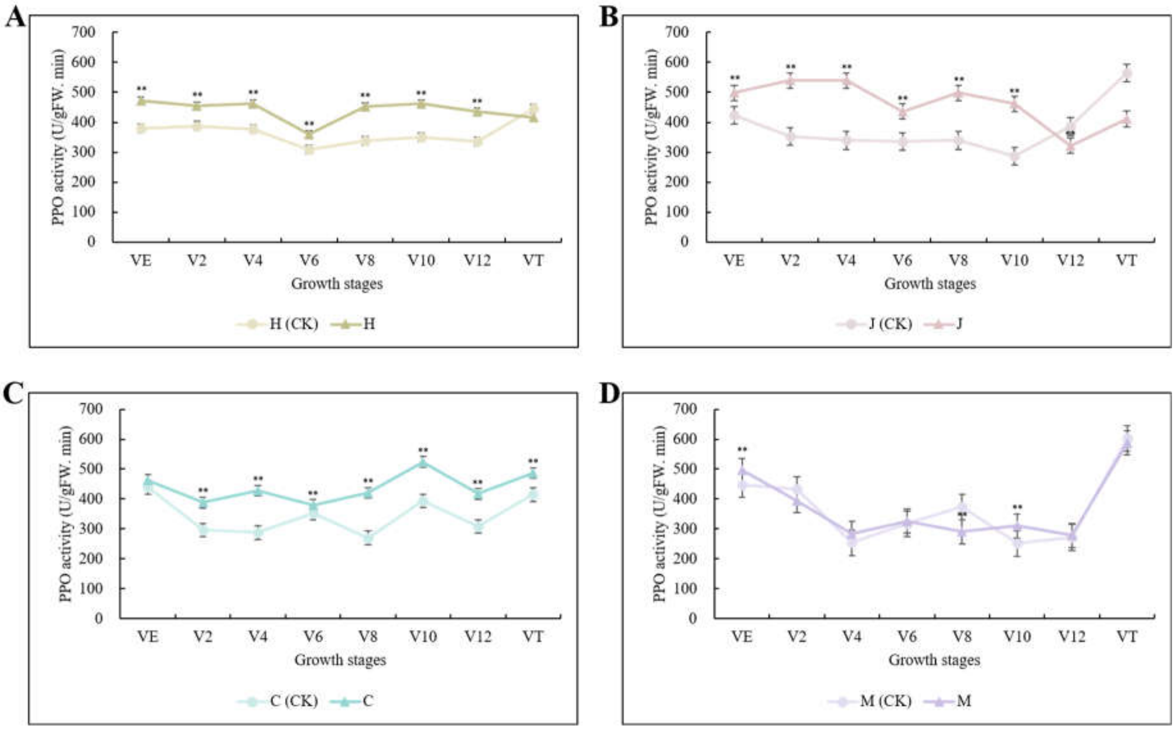

Figure S3. Changes of PPO activitiy in three typical maize inbred lines and disease-resistant control inbred line Mo17 at different growth stages Note: A: H (CK) indicate Huangzao4 uninoculated plants; H indicate Huangzao4 inoculated plants; B: J (CK) indicate Jing7 uninoculated plants; J indicate Jing7 inoculated plants; C: C (CK) indicate Chang7-2 uninoculated plants; C indicate Chang7-2 inoculated

plants; D: M (CK) indicate Mo17 uninoculated plants; M indicate Mo17 inoculated plants; “**” indicates $p<0.01$.

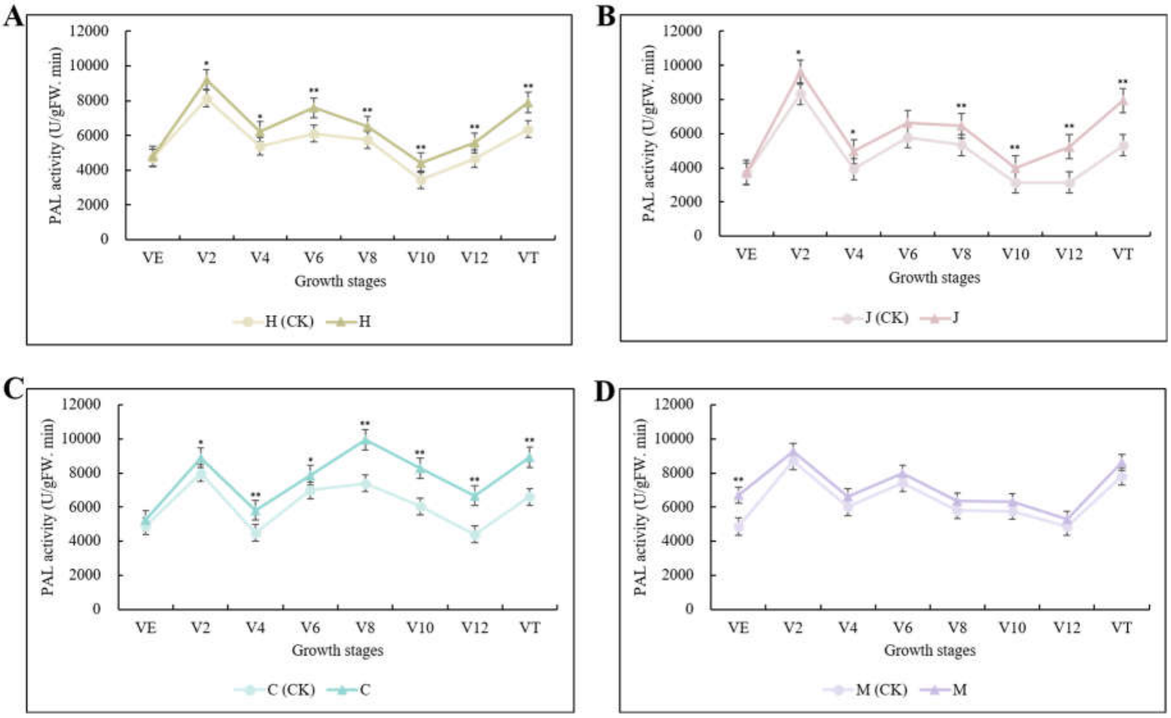

Figure S4. Changes of PAL activitiy in three typical maize inbred lines and disease-resistant control inbred line Mo17 at different growth stages Note: A: H (CK) indicate Huangzao4 uninoculated plants; H indicate Huangzao4 inoculated plants; B: J (CK) indicate Jing7 uninoculated plants; J indicate Jing7 inoculated plants; C: C (CK) indicate Chang7-2 uninoculated plants; C indicate Chang7-2 inoculated plants; D: M (CK) indicate Mo17 uninoculated plants; M indicate Mo17 inoculated plants; “*” indicates $p<0.05$; “**” indicates $p<0.01$.

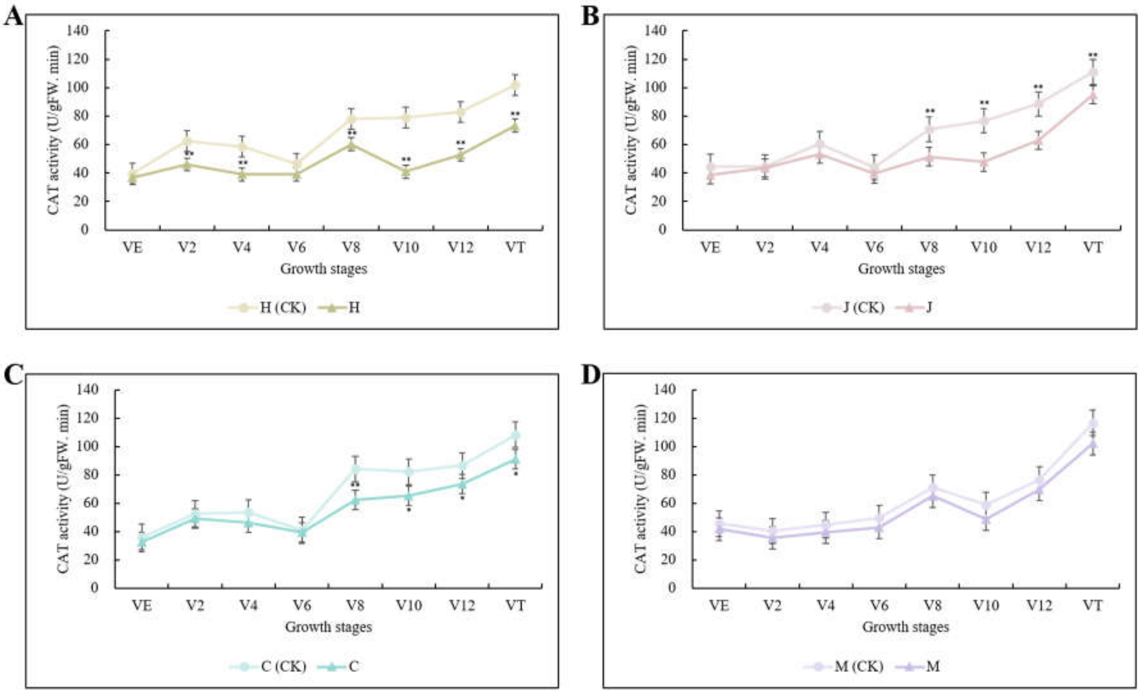

Figure S5. Changes of CAT activitiy in three typical maize inbred lines and disease-resistant control inbred line Mo17 at different growth stages Note: A: H (CK) indicate Huangzao4 uninoculated plants;

H indicate Huangzao4 inoculated plants; B: J (CK) indicate Jing7 uninoculated plants; J indicate Jing7 inoculated plants; C: C (CK) indicate Chang7-2 uninoculated plants; C indicate Chang7-2 inoculated plants; D: M (CK) indicate Mo17 uninoculated plants; M indicate Mo17 inoculated plants; "*" indicates $p<0.05$; "**" indicates $p<0.01$.
